# Oat Meal

**Published:** 1874-04

**Authors:** 


### OAT MEAL.

The oat meal is among the richest of all the grains in human nutriment, and as it thrives on poorer and colder soils than wheat, it ought to be the cheapest as it is the healthiest of all sorts of corn. If the husks are taken from the oats, and they are broken into small pieces, the product is called "groats," as Indian corn broken into small pieces is called "hominy." Oat meal is made by grinding the oats fine. This meal is boiled in water and makes "porridge" or oat meal mush. When baked we have "oat cakes." Both are used with butter, sugar, milk or molasses. Oat meal porridge with milk makes an exceedingly nutritious meal, having largely the elements of warmth and strength. To make good porridge the meal should fall through the fingers into boiling water, the whole amount in the course of a minute or two, so that all the meal should be equally cooked, and it should be stirred well all the time while boiling, so as to have no lumps and to avoid scorching. The longer it is boiled the better—at least an hour. Whey or milk may be used in boiling instead of water. If boiling water is poured on oat meal and stirred well the product is called "brose;" in this case the meal is raw and the mass is mushy, and is a coarse article of food. Still, with milk it is very nutritious and sustaining to hard working men.

To make the best oat meal cake stir the meal into cold water, knead most thoroughly, the more the better, roll it out thin, the thinner the better, and bake on an iron plate; to be eaten while hot, as it gets soggy after cooling.

Oat meal and milk make an amply sufficient breakfast for growing children up to ten years of age, and for all sedentary persons and many who live indoors. In a very few cases it causes discomfort after eating, then it should be avoided for the present. Oat meal cooked with garlic, leeks or onions, makes a very nutritious and wholesome meal. Some cook it with cabbage, turnip tops or other form of "greens," in the spring. This is a favorite dish in Wales, while the old Romans delighted in the combination of meal with "porrum" or leek, and from which "porridge" seems to have taken its name. A tablespoonful of raw oat meal stirred into a glass of cold water makes a safe and nutritious beverage for harvesters in summer. By keeping the system open, it cools it and carries off feverishness.

Oat meal has seventy-five per cent. of nutriment. Roast beef twenty-five.

